# Mineral Content and Phytochemical Composition of Avocado var. Hass Grown Using Sustainable Agriculture Practices in Ecuador

**DOI:** 10.3390/plants12091791

**Published:** 2023-04-27

**Authors:** William Viera, Pablo Gaona, Iván Samaniego, Andrea Sotomayor, Pablo Viteri, Michelle Noboa, Jorge Merino, Paúl Mejía, Chang Hwan Park

**Affiliations:** 1 Fruit Program, Tumbaco Experimental Farm, Instituto Nacional de Investigaciones Agropecuarias (INIAP), Av. Interoceánica Km. 15 and Eloy Alfaro, Quito 170902, Ecuador; andrevero1@hotmail.com (A.S.); pablo.viteri@iniap.gob.ec (P.V.); michellenoboa@gmail.com (M.N.); jorge.merino@iniap.gob.ec (J.M.);; 2 KOPIA Center Ecuador, Panamericana Sur Km. 1, Cutuglahua 171107, Ecuador; juanpaiq@hotmail.com (P.G.); park6725@gmail.com (C.H.P.); 3 Nutrition and Quality Department, Instituto Nacional de Investigaciones Agropecuarias (INIAP), Panamericana Sur Km. 1, Cutuglahua 171107, Ecuador; ivan.samaniego@iniap.gob.ec

**Keywords:** antioxidant activity, carotenoids, flavonoids, fatty acids, polyphenols, proximal analysis, zinc

## Abstract

Avocado demand has increased in recent years due to the nutraceutical properties that this fruit has and its positive impacts on human health; however, avocado production also requires sustainable alternatives to improve its cultivation. The objective of this study was to carry out characterization of the mineral content and phytochemical compounds in avocado fruit of the Hass variety grown using sustainable agricultural practices in Ecuador. Our results show an increase in fruit quality traits, such as firmness, and in the content of soluble solids, protein, fiber, fat, carotenoids, Ca, Mg, Zn and stearic acid in the pulp of the avocado Hass variety, as well as an initial trend of yield increase with the application of sustainable practices. Moreover, antioxidant activity was associated with polyphenol content. There were positive correlations of Mg with K and Ca, and of flavonoids with linolelaidic, linoleic and linolenic acids. Overall, our results indicate that avocado can be used as a functional and nutritional food due to its phytochemical composition and the mineral content of its pulp, which contributes to the promotion of its consumption and encourages healthy eating. In addition, the use of sustainable practices, such as fertigation and the application of microorganisms, is also promoted for growing avocado.

## 1. Introduction

Avocado (*Persea americana* Mill.) is a fruit crop that is undergoing continuous expansion in America, mainly in countries such as México, Colombia, Ecuador, Peru, Chile Venezuela, Bolivia and the Unites States of America [1]. Avocado consumption has undergone an annual increase of 1.4% [2] and is estimated to range from 1.05 to 3.50 kg per capita, which is considered high demand; thus the worldwide avocado market is expected to increase at an annual rate of 6.2% from 2017 to 2027 [3]. Moreover, the projected annual growth rate of the international Hass avocado market from 2018 to 2026 is 5.9% [4].

In Ecuador, there are around 7000 ha of avocados [5], but in recent years, farmers have had a tendency to increase the cultivated area, especially for the Hass variety [6]. Hass is the most cultivated avocado variety because it is in high demand in international markets [6], and also due to its high fruit yield, fruit availability during most of the year, and fruit resistance to postharvest management, its excellent fruit characteristics, such as a soft-creamy texture, high pulp content, high oil content, high amounts of phytochemicals and nutrients, as well as its agreeable aroma and taste [7]; therefore this variety is appreciated by consumers around the world due to its rich flavor, high overall quality and health-associated properties [8]. 

Issues related to sustainable avocado production should be carefully monitored [3]; therefore, the application of technologies to the sustainable production of this fruit crop is essential to improve productivity and fruit quality.

The application of plant nutrients via fertigation is a cost-effective and efficient method for the application of fertilizers that can improve productivity and decrease environmental pollution in comparison to conventional methods [9]. In addition, this kind of irrigation system trend enhances water use efficiency, reducing water consumption in crop production [10]. Avocado crops are mainly irrigated in Ecuador via flooding, and only a few orchards have irrigation system. It has been reported that fertigation has a positive effect on vegetative growth in avocado [11,12], and it has been mentioned that it can influence fruit quality in crops such as avocado [13] and mango [14].

Another sustainable practice is the use of microorganisms for crop production, and this approach is being promoted in Ecuador [15]. Some studies have reported the benefits of the application of fungi, such as Trichoderma, and mycorrhizae to the avocado crop, which mainly improve plant nutrient absorption [16,17,18]; while there is no information about their relationship with avocado fruit quality, it has been found that they can influence this characteristic in other fruit crops [19]. 

Avocado fruit demand has increased due to its functional compounds; it has beneficial effects on human health, because this fruit has great quantities of monounsaturated fatty acids and antioxidants [20]. Current research suggests that this fruit may have some protective effects against conditions such as diabetes, cardiovascular diseases and cancer, which are considered health problems in many countries around the world [21]. The avocado is a high-value fruit due to its mineral content [22,23]. It has been found that the pulp contains high amounts of potassium (K), magnesium (Mg), phosphorus (P) and calcium (Ca) [24,25]. This is beneficial to human health because K and Mg help to reduce hypertension and prevent cardiovascular diseases, while Mg helps to reduce blood pressure, the severity of asthma and migraine attacks [26,27]; P is involved in several biological processes; and Ca is essential to the appropriate functioning of the human body [28]. This fruit also contains bioactive compounds such as phenols and carotenoids [7], which are beneficial because they improve insulin sensitivity, decrease blood pressure, and have anti-inflammatory properties [29]. Consuming foods rich in phenols has been related to the prevention of Alzheimer’s disease [30]. Carotenoids are effective deactivators of free radicals in human cells, which prevents the development of some degenerative diseases, including cancer [21]. In addition, avocado contains unsaturated fatty acids such as linoleic, palmitoleic, linolenic acids, which represent up to 60% of the total fatty acid content in this fruit [21]. The consumption of oleic acids contributes to reducing blood pressure, preventing body weight increase, and controlling the development of hyperglycemia [31,32]. 

The health benefits of avocado fruit have been attributed to its high content of bioactive compounds [21]; therefore, the objective of this study was to carry out characterization of the mineral content and phytochemical compounds of avocado fruit of the Hass variety grown using sustainable agricultural practices in Ecuador. These results will contribute to knowledge of the value of this fruit as a nutraceutical and functional food source and promote avocado consumption due to its positive benefits for human health. In addition, this type of information has not been reported in Hass avocado grown under Ecuadorian climatic conditions.

## 2. Results

### 2.1. Trichoderma Population in the Soil

The colony-forming units (CFUs) per g of soil were estimated to quantify the variation in the Trichoderma populations. The initial Trichoderma population in the experimental plot was 3 × 10^2^ UFC g^−1^ prior to the inoculation. After one year of monthly inoculation, prior to fruit harvest, it was observed that the plots where *T. asperellum* was inoculated showed a slight increase to 4 × 10^3^ and 3 × 10^3^ UFC g^−1^ in the treatment with microorganisms and in the treatment with a combination of fertigation and microorganisms, respectively. Meanwhile, the treatment with fertigation and the control showed a slight decrease to 2 × 10^2^ and 1 × 10^2^ UFC g^−1^, respectively. 

### 2.2. Fruit Traits and Yield

The basic fruit traits and yield are shown in Table 1. There were no statistically significant differences in the productive traits; however, we observed a trend of increasing yield under the sustainable practices, especially the treatment that combined fertigation and microorganisms. The latter treatment also showed the highest firmness (2.23 N) and soluble solids (9.51 °Brix); in addition, it was observed that the treatment with fertigation shared its range of signification with 9.49 °Brix. 

### 2.3. Proximal Analysis

In the proximal analysis, protein, fiber and fat showed statistically significant differences among the practices evaluated (Table 2). The dry matter ranged from 29.31 to 32.24%, moisture content from 67.76 to 70.69%, ash from 8.90 to 10.44 g 100 g^−1^ and total carbohydrates from 3.55 to 4.67 g 100 g^−1^. Protein (range: 5.13 to 6.49 g 100 g^−1^) and fiber (range: 7.88 to 8.37 g 100 g^−1^) content was higher in the avocado pulp obtained from fruit grown using sustainable practices. Fat content was higher (72.66 g 100 g^−1^) in the treatment that combined fertigation and microorganisms.

### 2.4. Mineral Content

In terms of macroelements, Ca and Mg showed statistically significant differences, while only zinc (Zn) showed a significant difference among the microelements. Pulp obtained from the fertigation treatment showed high Ca (54.67 mg 100 g pulp^−1^), Mg (96.90 mg 100 g pulp^−1^) and Zn (2.43 mg 100 g pulp^−1^). The combined treatment and that with microorganisms also showed high Ca (36.03 and 31.10 mg 100 g pulp^−1^), whereas the treatment with microorganisms also showed high Zn (1.95 mg 100 g pulp^−1^). For the rest of the elements, all treatments showed similar values (Table 3). In all cases, K showed the highest percentage in the pulp, while manganese (Mn) showed the lowest percentage.

### 2.5. Antioxidant Compounds and Antioxidant Activity

Only carotenoid content showed statistically significant differences among the antioxidants compounds (Table 4). The polyphenol content ranged from 8.74 to 10.25 mg GAE g^−1^, while the flavonoid content ranged from 3.54 to 3.89 mg catechin g^−1^. All treatments related to sustainable practices obtained higher carotenoid content than the control, highlighting the effectiveness of fertigation with 52.60 µg β carotene g^−1^. 

In terms of antioxidant activity (AA), it was similar for all treatments (Table 3). AA ranged from 195.61 to 209.37 µmol TE g^−1^ using the ABTS method, whereas it ranged from 131.04 to 148.19 µmol TE g^−1^ using the FRAP method. 

### 2.6. Fatty Acids

In terms of fatty acid content, only palmitic and stearic acid showed statistically significant differences. The treatment using microorganisms obtained the highest content of palmitic acid (20.04%), while fertigation showed the highest stearic acid content (0.55%). The other fatty acids were present in similar amounts in the avocado pulp for all treatments (Table 5). The most predominant fatty acid was oleic acid (content around 50%), followed by palmitic acid (around 19%) and linoleic acid (around 12%), while the fatty acid present in the lowest proportion was stearic acid (around 0.5%).

### 2.7. Principal Component Analysis

**Figure 1:**
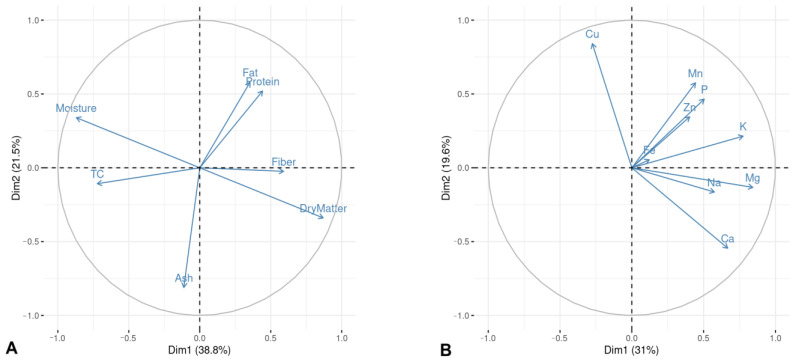

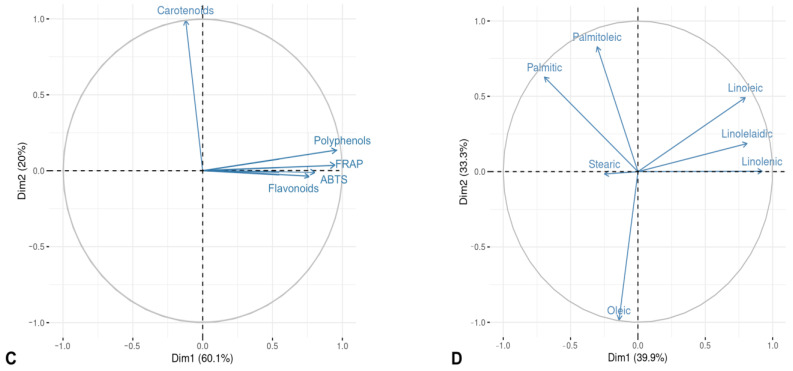
PCA analysis: (**A**) proximal variables, (**B**) minerals, (**C**) antioxidant compounds, and (**D**) fatty acids.

The results of the principal component analysis (PCA) for the chemical variable are shown in Figure 1. In terms of the proximal variables, the first two components explained 58.0% of the variance observed in the data. The first component shows contrast between moisture and total carbohydrates vs. dry matter, while the second component shows contrast between fat and protein vs. ash. Regarding minerals, the two components explained 50.6% of the variance; the first component shows contrast between Mg and K vs. Cu, whereas the second component shows contrast between Cu and Ca. For the antioxidant compounds, the two components explained 80.2% of the variance; the first component shows contrast between polyphenols and carotenoids, while the second component shows contrast between carotenoids and flavonoids. Finally, for the fatty acids, the two components explained 73.4% of the variance; the first component shows contrast between palmitic and linoleic acid, whereas the second component shows contrast between palmitoleic and oleic acid.

### 2.8. Correlation Analysis

The correlation values between the proximal variables and mineral elements are shown in Table 6. Positive correlations were obtained between ash and P (0.62), and Mg was positively correlated with Ca (0.65) and K (0.74). Meanwhile, a negative correlation was observed between dry matter and moisture (−0.99); fat was negatively correlated with total carbohydrates (−0.58), Na (−0.58) and Zn (−0.54); and protein was negatively correlated with Fe (0.59), and Ca and Cu (−0.56). 

In terms of the correlation between antioxidant compounds and fatty acids (Table 7), there polyphenol content was positively correlated with FRAP (0.93), ABTS (0.73), flavonoids (0.63) and linolelaidic acid (0.50); flavonoid content was positively correlated with linolelaidic acid (0.54), linoleic acid (0.50) and linolenic acid (0.60); palmitic and palmitoleic acid were positively correlated (0.58); linolelaidic and linoleic acid were positively correlated (0.59); and linolenic acid was positively correlated with linolelaidic acid (0.58) and linoleic acid (0.79).

Meanwhile, a negative correlation was observed between flavonoid content and palmitic acid (−0.51); palmitic acid was negatively correlated with oleic acid (−0.55), linolelaidic acid (−0.59) and linolenic acid (−0.59); and oleic acid was negatively correlated with palmitoleic acid (−0.73) and linoleic acid (−0.62). 

## 3. Discussion

Avocado has recently been categorized as a “superfood” due to its exceptional nutritional composition, antioxidant compounds and biochemical profile, which has turned it in a popular fruit in the market [33]. It is evident that avocado demand and production are increasing, and the economic market for avocados continues to expand globally [3]. The variety most grown around the world is Hass avocado due to its high fruit yield, resistance to postharvest management, fruit availability almost year-round, and excellent fruit quality characteristics [7]. In Ecuador, the technification of the avocado crop is limited, and several farmers continue to grow this fruit the conventional way due to a lack of knowledge on technologies in cultivation that can be applied to this fruit crop [34]. The main form of avocado cultivation is monoculture. This type of agriculture is related to high water usage (flooding irrigation); in addition, monoculture production causes other environmental impacts due to the excess use of inorganic fertilizers [3]. For this reason, practices such as fertigation and microorganisms for avocado cultivation constitute alternatives that can reduce the environmental impacts of avocado production and provide the benefits of avocado plant nutrition [11,12,16,17,18]; however; there is scarce information about their relationships with fruit quality parameters. 

*Trichoderma asperellum* contributes to improving nutrient absorption in avocado plants [17]. The *T. asperellum* population obtained after one year of inoculation in the plots where the fungus was applied was slightly larger than that obtained in the plots where there was no inoculation. The results of the inoculation follow the trend found by Viera et al. [35] in blackberry orchards inoculated with *T. asperellum*, which means that this practice helps to increase the population of this fungus in the soil, as occurred in the treatments with microorganisms and the combination of fertigation and microorganisms. Nevertheless, the latter authors mention that for the population in the soil to be considered stable, a theoretical concentration of 1 × 10^4^ must be reached; consequently, the inoculation of microorganisms must be continuous, especially in this kind of perennial fruit crop. On the other hand, the populations of Trichoderma found in the treatments with fertigation and the control occurred due to the presence of native strains in the soil, and their variation might be influenced by environmental factors. Regarding *Glomus iranicium*, it has been stated that this mycorrhizae fungus establishes a symbiotic relationship with avocado roots, and also influences the nutrient uptake of the plant [17]. Therefore, plant nutrition has an essential role in determining fruit quality. 

### 3.1. Fruit Traits and Yield

The fruit weights obtained for all treatments were similar to those (241.39 to 255.22 g) reported by Salazar-García et al. [36], but these authors also found higher values (266.54 g) depending on the site on which the avocado was grown. In terms of yield, although there were no statistically significant differences, we observed an increasing trend in this trait with the use of the sustainable practices; this tendency has been found in other fruit crops with the application of microorganisms [35]. This increase in yield was associated with the fact that the practices also enhanced the number of fruits per plant; therefore, the total yield was also increased. This increment in fruit number may be related to a better fruit set due to the precise fertilization and the nutrient absorption improvement [37]. 

Fruit firmness reached a value similar to that (around 1 N) reported by Cho et al. [38] after 8 days of storage; on the other hand, the soluble solids obtained a similar value to that (9.50 °Brix) reported by El-Moniem et al. [39] in avocado fruit harvest from an orchard treated with drip irrigation. It was observed that the combined treatment showed the highest values of firmness and soluble solids; this might be related to the optimization of fertilization by the fertigation system, especially the effect of Ca, an element that was highlighted in the combined treatment, and the enhancement of plant nutrition produced by the microorganisms, which would influence both parameters [37,40]. 

### 3.2. Proximal Variables

Moisture is one of the most important indices evaluated in foods, especially fruits. It is a good indicator of their economic value because it reflects the fruits’ solid contents and serves to assess their perishability [41]. The moisture percentage found in this study was similar to that (around 70%) reported by Catañeda-Saucedo et al. [42] for avocado var. Hass, while the dry matter was higher than the value (24.60%) reported by Ramos-Aguilar et al. [7], but similar to the percentage (31.09%) found by Ozdemir and Topuz [43]. 

Fiber, protein and fat content were higher in the treatment that combined fertigation and microorganisms; these results were higher than the values (6.25 g 100 g^−1^ for the two first parameters and 71.34 g 100 g^−1^ for fat) reported by Castañeda-Saucedo et al. [42] in avocado orchards with conventional irrigation in Mexico. Similarly to this study, a positive influence of the use of a fertigation system on avocado fruit quality traits was found by Yar Narváez [13]. It is certain that avocado fruits have higher results for these traits because their consumption is related to the reduced risk of diseases, such as cardiovascular issues, and improved digestion, as in the case of fiber. 

The increase in protein content resulting from the application of the combined treatment could be associated with the fact that proteins are formed of N (16%), and the fertigation system avoids the loss of N via lixiviation [44]; moreover, also has better plant absorption due to the effects of microorganisms [17]. On the other hand, fiber content could be influenced by the high content of Ca reached using sustainable practices. A high fat content in avocado fruit requires adequate nutrient content, especially K [45]; thus, sustainable technologies would promote this characteristic in the fruit. 

Castañeda-Saucedo et al. [42] reached an ash content of 10.40 g 100 g^−1^ in an orchard with conventional management, which is similar to the value of the control (without fertigation and microorganisms) used in this study. The total carbohydrate results from this study were higher than the value (3.29 g 100 g^−1^) reported by Santos et al. [46] in Hass avocado and by Tan et al. [47] in Red Thompson avocado (3.39 g 100 g^−1^); this is good because it is recommended that people consume calories in the form of carbohydrates.

### 3.3. Mineral Content

In this research, the minerals that showed statistically significant differences were Ca, Mg and Zn. These three nutrients have beneficial effects on human health, and their consumption through fruits is recommended. Martínez-Ballesta et al. [48] mention that Ca concentration in plant foods has a broad range of variation, and it is an indispensable mineral for human health; it enhances the biological functions of tissues, acts as a cofactor for enzyme reactions and intervenes in physiological processes. Mg has a strong presence in plant foods, and also plays an essential role in human body maintenance; it is associated with energy metabolism, enzyme cofactors and the prevention of chronic disease. The same author states that Zn concentration varies in plant foods, but fruits are a good source of this element; it is needed for enzyme structure and activity. In addition, its consumption improves the immunity system and it may be a promising option for producing immunity against COVID-19 [49]. The highest amounts of these elements were obtained in the treatment with fertigation, and this effect of increased mineral content in the fruit was also found by Yar Narváez [13]. This effect might be related to the fact that fertigation is a system that allows for fertilization to be directed to the root area, making plant nutrition more efficient and improving plant nutrient intake; and this may also be reflected in the pulp mineral content. 

The Ca and Mg results of this study were higher than those (10.00 and 41.00 mg 100 g^−1^) reported by Ortega Tovar [22] for Hass avocado. The highest concentration of Ca obtained in the treatment with fertigation was similar to that (54.90 mg 100 g^−1^) found by Morais et al. [50] in avocado grown in Brazil, but the percentages of Mg and Zn were higher than the values (82.1 and 1.20 mg 100 g^−1^) reported by the last authors. The Zn value was also higher than that (0.68 mg 100 g^−1^) mentioned by Araújo et al. [51]. 

In terms of the other macronutrients, the results of K and P were higher than those (463.00 and 40.00 mg 100 g^−1^, respectively) found by Ortega Tovar [22]. The percentages of P and K were higher than those that reported by Morais et al. [50], with 1195.20 mg 100 g^−1^ for P, and by Araújo et al. [51], with 54 mg 100 g^−1^ for K. Meanwhile, regarding the micronutrients, sodium (Na), cooper (Cu) and iron (Fe) concentrations were higher than those found by the latter author (4.00, 0.35, 1.06 mg 100 g^−1^, respectively), but Mn was lower (2.30 mg 100 g^−1^). On another hand, the percentages of Na, Cu, Fe and Mn were lower than the values (32.30, 1.30, 2.70 and 1.30 mg 100 g^−1^, respectively) reported by Morais et al. [50].

K was the mineral found in the highest proportion in the avocado pulp of Hass avocado, whereas Mn was found in the lowest proportion, as also reported by Morais et al. [50] and Araújo et al. [51]; nevertheless, Fe was found in a lower percentage in the pulp in another study [22]. 

### 3.4. Antioxidant Compounds 

Antioxidant compounds present in food are appreciated because they can delay, inhibit or control the oxidation process in the cells [52]. An example of this type of compound the polyphenols, which are important from a nutritional viewpoint and are also a commercial factor [53], with the fruits being a great source of these compounds [54]. There were no differences in polyphenol content in the Hass variety among the treatments evaluated in this study; however, all values were higher than those reported by Husen et al. [55], Amado et al. [56] and Lyu et al. [57] in the Hass variety (0.94, 2.04 and 0.20 mg GAE 100 g^1^, respectively). 

Different health properties, such as antiallergic, antiviral, anti-inflammatory, therapeutic and anticarcinogenic properties, have been attributed to flavonoids [58]. All flavonoid values were higher than those (2.37 mg catechin g^−1^) reported by Sellamuthu et al. [59] for Hass avocado. Lyu et al. [57] reported that avocado pulp has good polyphenol and flavonoid content. 

Carotenoids such as β-carotene are a precursor to vitamin A; these compounds have been associated with stimulation of the metabolism and the immune system, and to the prevention of degenerative diseases [60]. All of our results showed higher values than those (28.9 µg β carotene g^−1^) reported by Monje-Rojas and Campos [61] for the Hass variety. However, the contents obtained where the practices were applied were higher than the control. It was observed that the treatment with fertigation led to the highest content of carotenoids; this could be related to the fact that it provided soluble elements directly to the root area and with a more continuous frequency, which meant that they were more assimilated by the plant, and this nutrition is reflected in the fruit quality. The same trend was reported by Yar Narváez [13] for the varieties Hass and Fuerte. In fact, it has been reported that fertilization influences the increase in the content of antioxidant compounds [62]; thus, the use of fertigation will optimize fertilizer application to improve this fruit trait. 

### 3.5. Fatty Acid Composition

Regarding fatty acids, palmitic and stearic acids are the predominant saturated fatty acids among the interesterified fats, and they have been related to cholesterol levels in the body [63]. The results of the treatment with microorganisms showed that the palmitic acid level was higher than that obtained by Ramos-Aguilar et al. [7], who reported a value of 2.90%; however, this author also found a higher stearic acid percentage (0.64%) than the value reached through fertigation treatment, which was the highest in this study. It has been reported that stearic acid might attenuate pulmonary fibrosis, supporting the alleviation of this disease [64]. 

In avocado, the predominant fatty acid is oleic acid, followed by palmitic acid [7], which is in agreement with the results of this research. Oleic acid has beneficial effects for human health, such as reducing bad cholesterol levels [65] and reducing huger, and thus, food consumption, which also means avoiding obesity [66]. The content of this fatty acid was higher in the avocado fruit pulp, as has also been reported in other studies [7,42].

The results for palmitic, palmitoleic, oleic and linoleic acids were higher than those reported by Castañeda-Saucedo et al. [42] (14.10, 5.39, 44.07 and 7.46%, respectively); it was observed that in most cases, the treatments that applied the sustainable practices showed the highest values for these fatty acids. In comparison to this study, Ortega Tovar [22] found lower percentages of palmitic (13.76%) and palmitoleic (5.98%) acids than those found in this research; however, they observed higher concentrations of stearic (1.48%), oleic (64.87%) and linolenic (2.52%) acids, and a similar amount of linoleic acid (11.43%). 

On the other hand, Yanty et al. [67], who examined fatty acids using oil from the Hass variety, and Pedreschi et al. [8], who used the fruit of an African avocado variety, found a lower content of palmitic and palmitoleic acids (less than 15 and 5%, respectively), and the highest percentage of oleic, linoleic and linolenic acids (more than 63, 14 and 1%, respectively). 

Finally, data on linolelaidic acid has not been reported in avocado studies; however, it has been found in low percentages in another tropical fruits, such as durian [68]. The percentage of this acid was low in comparison to the main fatty acid of Hass avocado (oleic acid), and it was also lower than that reported in durian (12.39%). This trans fatty acid is mainly found in processed food; however, it is an isomer of linoleic acid, and the sample extraction process can slightly increase its concentration.

### 3.6. Principal Component Analysis 

The PCA results show that ash content had a clear contrast with the rest of the proximal variables, except fiber. This might be associated with the fact that ash content is related to the mineral concentration [69], unlike the other parameters. In terms of minerals, Morais et al. [50] assessed avocado fruit grown in Brazil, and they found that K and Ca showed contrast with the other elements; nevertheless, in this study, the element that showed contrast with the rest was Cu; this could be related to the fact the response might vary depending on the cultivation conditions. In addition, avocado pulp showed high amounts of K and Ca, as was the trend in the study of the latter authors. Regarding antioxidant compounds, polyphenol content was associated with AA, which agrees with the results of Lyu et al. [57]; nevertheless, an influence of flavonoid content also was observed in this analysis. We found a clear contrast between carotenoids and the rest of the antioxidants, and its proportion was also higher, indicating that avocado fruit may constitute a good source for this antioxidant compound. Finally, oleic acid showed a clear contrast with the other fatty acids, which could indicate that the higher the oleic acid content, the lower the rest of the fatty acids; this contrast has also been found in other fruits, especially those with palmitic and linoleic acids [70]. This approach is positive because this fatty acid is recommended for intake due to its benefits associated with cholesterol reduction [65]. 

### 3.7. Correlation Analysis

There was a negative correlation between fat and carbohydrate content, which is related to the fact that the avocado fruit is rich in oil and fiber and low in sugars (simple carbohydrate). The amount of ash is associated with the mineral content in foods [69]; in this study, this parameter showed a positive relationship with P, a nutrient that is important in human consumption because it is required for energy [71]. Protein showed a negative correlation with Fe; however, the latter mineral is a trace element and it is not needed in high amounts in the human body [48]. Fat also showed negative correlation with sodium, which is not bad, since this fruit is appetizing to consumers due to its fat content (natural fat), and the Na intake needed by the human body is very small [48]; moreover, fat also showed a negative relationship with Zn, an element which is also important in a healthy diet. On the other hand, Mg was highly correlated with Ca and K; these relationships are positive from the point of view of human health because the consumption of these minerals is beneficial to the body’s metabolism and functionality [40]. On the other hand, a negative correlation was observed between Ca and Cu; however, the latter element is required in low amounts for body to function [48].

Polyphenol content showed a correlation with flavonoid content, a result that is similar to the trend reported by Lyu et al. [57], who found a positive correlation of 0.40. The same authors reported a high correlation (0.91) between polyphenol content and AA (ABTS), a result similar to that found in this study. Flavonoids showed a positive correlation with linoleic and linolenic acids, which is adequate because these compounds are associated with the prevention of cardiac diseases [31,58]; in addition, both fatty acids were also positively correlated. On the other hand, oleic acid showed a negative correlation with palmitic, which is acceptable because the former helps to reduce bad cholesterol levels [65]; however, the former is associated with an increase in this type of cholesterol [63], especially when it replaces carbohydrates or other types of fats in the diet.

### 3.8. Final Remarks

The demand for avocado fruit is continuously increasing due to its nutraceutical benefits to human health, and avocado production is mainly carried out in countries outside of large consumer markets; therefore, the promotion of sustainable practices for its production is required nowadays. In addition, it would be valuable to offer sustainably produced avocados to consumers [3]. On the other hand, as most avocado orchards are produced based on intensive monoculture, several issues associated with environmental complications and natural resource use are involved in this type of cultivation system. The promotion of alternatives, such as fertigation due to its efficiency in the use of water and located fertilization [72], and the use of microorganisms because of their influence on plant nutrient absorption [17], are adequate for achieving sustainable production because the adoption of technological innovation encourages better agri-food systems [73]; therefore, there is a need for the sustainable production of avocado. 

Finally, these findings contribute to showing the good fruit quality of Hass avocados grown under the environmental conditions of Ecuador using sustainable practices that would lead to a better production system, and attaining a healthier product for the consumers. However, it has to be considered that fruit quality traits can be influenced by climatic conditions and agronomic management at other sites of avocado production. 

## 4. Materials and Methods

### 4.1. Experimental Site and Plant Material

The study was carried out at the Nutrition and Quality Laboratory (ISO/IEC 17025) of the National Institute of Agricultural Research (INIAP), located in Pichincha, Ecuador (00°22’57″ S and 78°33’18″ W).

The fruits of the avocado Hass variety used for this research were harvested 36 weeks after flowering. Avocado Hass trees (grafted in local rootstocks from a Mexican cultivar) were sown every 4 m among plants and every 5 m among rows, and they were 3.5 years old. The experimental orchard is located in the Tumbaco Experimental Farm of INIAP, located in Tumbaco (Pichincha, Ecuador); this site is located at 2348 masl, latitude 0°12′57″ S, longitude 78°24′43″ W, and has annual precipitation of 892 mm, an average temperature of 17 °C, average relative humidity of 71% and an accumulated heliophany of 2039 h per year. 

### 4.2. Agronomical Management (Sustainable Agronomic Practices)

The sustainable practices used in the experimental plots were fertigation, to provide nutrients to the plants, and the application of microorganisms to the soil (*T. asperellum* and *G. iranicum var. tenuihypharum*). There was a control plot without the use of fertigation (edaphic fertilization) and without the application of microorganisms. Additionally, pruning (an environmentally friendly practice) was applied to all the trees on the experimental plot. The plants receive formation pruning (pyramidal growing) after 6 months of transplanting, and then, maintenance pruning was performed every 6 months [74]. 

All experimental plots had dropping irrigation systems for supplying water. Each tree received 16 L dia^−1^, divided in two irrigation times per day (8 L in the morning and 8 L in the afternoon), five times per week. 

The fertilization was set based on soil analysis and crop requirements. A nutritive solution was prepared for the fertigation, and it contained the following macroelements: N: 120 kg ha^−1^, P: 19 kg ha^−1^, K: 167 kg ha^−1^, Ca: 99 kg ha^−1^, Mg: 27 kg ha^−1^ and S: 40 kg ha^−1^; additionally, it contained the following microelements: Fe: 1.2 kg ha^−1^, B: 2.9 kg ha^−1^, Mn: 0.7 kg ha^−1^, Zn: 3.9 kg ha^−1^ and Cu: 1.4 kg ha^−1^. For the control, edaphic fertilization was applied (fractioned three times per year); it was estimated to reach similar amounts of nutrients to the fertigation. We used 100 g plant^−1^ of a complete fertilizer (N: 15%, P_2_O_5_: 9%, MgO: 1.8%, SO_3_: 9.3%, Zn: 0.02%, B: 0.015%, K_2_O: 20%, S: 3.8% and Mn: 0.02%), 820 g plant^−1^ of calcium nitrate (N: 16%, CaO: 34%), 1150 g plant^−1^ of potassium nitrate (N: 13% and K_2_O: 44%) and 350 g plant^−1^ of magnesium sulfate (MgO: 25% and S: 20%). In the flowering stage, three foliar applications with a fertilizer containing Ca: 10.5% and B: 2% were carried out to avoid flower dropping.

In the case of the application of microorganisms, 250 mL of a solution (dose of 1 g L^−1^) of *T. asperrellum* (1.53 × 10^9^ conidia/g) was applied to four 20 cm deep holes around the plants. Regarding the mycorrhizae fungus, 7 g plant^−1^ was applied every three months; the inoculation was performed in holes (as described before) different to those in which the Trichoderma was applied. Microorganism application as carried out monthly, one year before the fruit harvest. In addition, a soil analysis was carried out before starting the Trichoderma applications and fruit harvest to determine the variation in the population of *T. asperellum* in the soil through the estimation of CFU per g of soil [35].

### 4.3. Fruit Traits and Yield

To determine the basic fruit traits, 30 fruits were evaluated for each treatment. Fruit weight was measured using a digital scale (BBL53, Boeco, Hamburg, Germany), and the results were expressed in grams. Firmness and soluble solid content were measured in fruits stored for 8 days at room temperature. The penetration force required to penetrate the avocado pulp was measured directly using a digital penetrometer (FR-5120, Lutron, Coopersburg, PA, USA), and the result was expressed in Newtons. The soluble solids were also estimated using a digital refractometer (BOE 32395, Boeco, Hamburg, Germany) and expressed in Brix degrees.

The yield was calculated using 10 plants from each treatment. In these plants, the number of fruits per plant was counted and the total fruit weight per plant was measured. 

### 4.4. Proximal Analyses

Chemical analyses were conducted on the avocado fruits at harvesting time. Proximal analysis was carried out using the methods described by AOAC [75]. Moisture content was obtained via gravimetry; an amount of 10 g of pulp was weighed and dried in an oven (Lab-Line IMPERIAL V, Cole-Parmer, Vernon Hills, IL, USA) at 105 °C for 16 h; the result was calculated by determining the difference in weight and reported as a percentage. The dry matter was calculated by determining differences in the percentages of humidity.

To determine the ash content, 1 g of each sample was weighed using porcelain crucibles with a 25 mL capacity, and subjected to a calcination process at 500 °C in a muffle (48000, Thermolyne, Dubuque, IA, USA) for 12 h. The calcined samples were cooled for 1 h and transferred to a desiccator; then, each crucible was weighed. The ash content was calculated by determining the difference in weight, and we expressed the results in g of ash per 100 g of pulp dry weight (DW).

In terms of protein content, 1 g of each sample was weighed in a 250 mL digestion tube, and 2 tablets of copper catalyst (3.5 g K_2_SO_4_ and 0.4 g CuSO_4_ × 5H_2_O) and 15 mL of concentrated sulfuric acid were added. The tubes were placed in a digestion block and heated at 400 °C for 1 h; they were cooled for 1 h and placed in a protein analyzer (Kjeltec model 8400, FOSS, Denmark, Hillerod) where distillation and titration were carried out, and the results were expressed as g of protein per 100 g of pulp (DW).

Fiber content was determined by weighing 1 g of each sample in porous glass crucibles (100 µm) and placing them in a fiber analyzer (Fibertec 8000, FOSS, Hillerod, Denmark); once the heater reached 120 °C, the samples were subjected to acid digestion (sulfuric acid 1.25% *v*/*v*) and alkaline digestion (NaOH 1.25% p/v) for 1 h. After this time, the samples underwent a washing process with distilled water; the crucibles with the digested samples were removed and placed in an oven (Lab-Line IMPERIAL V, Cole-Parmer, Vernon Hills, IL, USA) at 105 °C for 1 h. Finally, they were placed in a desiccator, cooled and weighed; we expressed the results as g of fiber per 100 g of pulp (DW).

Fat content was determined by weighing 2 g of each sample in stainless steel beakers and covering them with cotton. They were placed in a fat analyzer (SoxtecTM 2043, FOSS, Denmark, Hillerod); once the heater reached 130 °C, thimbles were submerged for 10 min and the fat extraction process was carried out for 30 min. The beakers were removed and placed in an oven (Lab-Line IMPERIAL V, Cole-Parmer, Vernon Hills, IL, USA) at 105 °C for 1 h in order to completely volatilize the hexane. Then, they were placed in a desiccator, cooled and weighed; we expressed the results as g of fat per 100 g of pulp (DW).

Total carbohydrate content was calculated by determining the difference between carbohydrates and the ash, protein, fiber and fat content; the results were expressed as g of total carbohydrates per 100 g of pulp (DW).

### 4.5. Mineral Analysis

Prior to the analysis, the pulp was dried using a lyophilizer (Labfreez, FD18MR, Wanchai, Hong Kong) and pulverized in a mill (Retsch, ZM 200, Hannoversch Münden, Germany); then, the particle size was made uniform by passing the dry sample through a stainless steel sieve (1 mm mesh).

A mineralization process was applied to the samples according to the method proposed by AOAC [75]. Thus, 1 g of the dry sample was incinerated in a muffle (model 48000, Thermolyne, Dubuque, USA) at 400 °C for 12 h. After this, the sample was cooled in a desiccator and passed to a heating plate (Witeg, Wertheim, Germany). We added 5 mL of HCL (37%) and 10 mL of Type I water (18.2 MΩ cm) to the sample and allowed it to digest at 100 °C until half of its volume had bene obtained. The sample was filtered in a 100 mL flask with qualitative filter paper (Watman, Maidstone, UK), and filled with Type I water.

For the determination of macronutrients, such as Ca and Mg, 0.5 mL of 1% lanthanum solution was added to 4.5 mL of the sample. Regarding Na and K, 0.5 mL of 1% lithium solution was added to the same volume of the sample. The absorbance was measured using an atomic absorption spectrophotometer (AA7000, Shimadzu, Tokyo, Japan). In the case of P, 4 mL of Type I water and 0.5 mL of ammonium molybdovanadate 1% solution were added to 0.5 mL of the sample. A UV–Visible spectrophotometer (UV2600, Shimadzu, Tokyo, Japan) was used to measure the absorbance. In terms of micronutrients (Zn, Cu, Fe and Mn), no solutions were added for interference. An amount of 5 mL of the sample was used. An atomic absorption spectrophotometer (AA7000, Shimadzu, Tokyo, Japan) was used to measure the absorbance. Quantification was carried out using calibration curves for each element, and the results were expressed as mg 100 g of pulp (DW).

### 4.6. Antioxidant Compound Analysis

The extraction of phytocomponents was carried out using the methodology detailed by Viera et al. [76], where 0.3 g of dry sample was mixed with 5 mL of methanol/water/formic acid solution (70:30:0.1 *v*/*v*/*v*). Then, FAST PREP 24 (MP Biomedicals, Fisher Scientific, Hampton, VA, USA) was used for the shaking extraction process (5 min), and then, an ultrasound bath (Cole-Parmer, Chicago, IL, USA) was used for 10 min. The sample was centrifuged using a 4–16 KS centrifuge (Sigma, Neustadt, Germany) for 10 min at 5500 rpm, and the supernatant was removed. This process was repeated three times, and the extract was also used for the AA. 

The polyphenol, flavonoid and carotenoid contents were measured using UV–visible spectrophotometry, according to the methodology detailed by Viera et al. [76]. For determining total polyphenol content, 1 mL of Folin–Ciocalteu reagent and 6 mL of distilled water were added to 1 mL of the diluted extract. After 3 min, we added 2 mL of 20% Na_2_CO_3_ (*w*/*v*), and the extract was warmed up to 40 °C for 2 min. A UV-VIS spectrophotometer (model 2600, Shimadzu, Kyoto, Japan) was used to measure the absorbance at 760 nm. Five extraction cycles were carried out, and quantification was performed through interpolation of the absorbance using a calibration curve made with 0 to 100 mg gallic acid L^−1^. The results were reported as mg of gallic acid equivalents per g of pulp (mg GAE g^−1^ DW).

To measure the flavonoid content, 1 mL of the diluted extract was mixed with 4 mL of distilled water. Then, 0.3 mL of 10% aluminum chloride (*w*/*v*), 0.3 mL of 5% sodium nitrite (*w*/*v*) and 2 mL of 1N NaOH were added; distilled water was added to obtain a final volume of 10 mL. A UV-VIS spectrophotometer (model 2600, Shimadzu, Kyoto, Japan) was used to measure the absorbance at 490 nm. Five extraction cycles were performed, and quantification was carried out through interpolation of the absorbance value of each sample using a calibration curve made with 0–100 mg catechin L^−1^. The results were reported as mg of catechin equivalents per g of pulp (mg catechin g^−1^ DW).

For measuring the total carotenoid content, 1 g of the dry sample was mixed with 50 mL of a solvent mixture of 50% hexane, 25% ethanol, 25% acetone (*v*/*v*/*v*), 0.1% butylated hydroxytoluene (BHT) (p/v) and 5 g calcium chloride (p/v). Using a refrigerated water bath (4 °C), the mixture was blended for 20 min. The separation phase was obtained after 10 min through the addition of 15 mL of distilled water. Then, the organic phase was mixed with 50 mL of hexane. A UV-VIS spectrophotometer (model 2600, Shimadzu, Kyoto, Japan) was used to measure the absorbance at 450 nm. The results were reported as µg of β carotene per gram of pulp (µg of β carotene g^−1^ DW).

### 4.7. Antioxidant Activity 

For the ABTS method, AA was measured using the 2,2-azinobis (3-ethyl-benzothiazoline-6-sulfonic acid) cation bleaching method (ABTS•+), as detailed by Viera et al. [76]. A UV-VIS spectrophotometer (model 2600, Shimadzu, Kyoto, Japan) was used to measure the absorbance at 734 nm. The AA was calculated by interpolating the absorbance using a calibration curve made with a Trolox standard (0 to800 µmol Trolox L^−1^). The results were expressed as µmol Trolox equivalent (TE) per g of pulp (µmol TE g^−1^ DW).

Additionally, the AA was also measured using the Ferric reducing power (FRAP) method, as detailed by Viera et al. [76]. A UV-VIS spectrophotometer (model 2600, Shimadzu, Kyoto, Japan) was used to measure the absorbance at 700 nm. The results were expressed as µmol TE per g of pulp (µmol TE g^−1^ DW).

### 4.8. Fatty Acid Analysis 

The determination of fatty acids was carried out using the method described by AOAC [75]. For the analysis, 0.05 g of fat was weighed in a 10 mL test tube; then, 1 mL of potassium hydroxide solution (0.5 M in methanol) was added, and the solution was heated in a boiling water bath for 10 min. Then, it was cooled, and 0.4 mL of a 4:1 *v*/*v* hydrochloric acid solution in methanol was added, and the solution was heated in a water bath for 25 min. The sample was cooled at room temperature (25 °C); then, 2 mL of bidistilled water and 3 mL of hexane were added, and the tube was shaken for 30 s in a vortex. The sample was left to rest to achieve separation of the organic and aqueous phases. The organic phase was recovered, and the procedure was repeated 3 times. The organic extract containing the fatty acid esters was concentrated to dryness using a stream of nitrogen at room temperature, redissolved with 2 mL of hexane (HPLC grade) and placed in a 2 mL amber vial for analysis using a gas chromatographer (7890 A, Agilent, Waldbronn, Germany) coupled to a flame ionization detector. The separation was carried out in a capillary column Supelco SP TM 2560 (100 m × 0.25 mm × 0.2 µm) using a column oven temperature setting of 140 °C for 5 min, and we increased the temperature by 4 °C min^−1^ up to 240 °C, using helium as a carrier gas. Identification was carried out by comparing the retention times of the peaks with their respective standards, for which a SIPELCO FAME MIX standard from C4 to C24 was used. Quantification was carried out using the external standard method, comparing the area of the sample with the area of its respective standard. The results were expressed as percentages.

### 4.9. Statistical Analysis

A total of 30 fruits were collected for each treatment to be dried, and constituted the sample (5 fruits per replication). The experimental unit was a 20 g sample of freeze-dried pulp. A randomized completed design with six replications was used for this research. R statistical software version 4.2.2. was used to carry out the data analysis. The normality of the data was assessed using the Shapiro–Wilk test, while the homogeneity of variances was determined using the Levene test. 

A one-way ANOVA function in R was used to carry out the univariate analysis, this function allows for heteroscedasticity of variances. A Tukey test at 5% was used to find differences between the means. However, the Kruskal–Wallis test was used to find statistically significant differences in the variables firmness, fat, protein, Ca, Cu, Fe, Mn, Zn and stearic acid, because they did not show normality of the residual experimental errors. 

Pearson correlation coefficients were estimated to identify the relationships between the proximal variables and minerals, and between the antioxidant compounds and the oleic acids. Finally, principal component analysis was applied to visualize the relationships among the variables.

## 5. Conclusions

The results of this study corroborate that avocado is a great source of essential elements, bioactive compounds and fatty acids. We observed an increasing trend for fruit traits, such as firmness, soluble solids, protein, fiber, fat, Ca, Mg, Zn, carotenoid content and palmitic and stearic acid in the pulp of Hass avocado following the application of sustainable practices such as fertigation and the use of microorganisms; this indicates that the technification of the crop helps to improve its fruit quality. In addition, an initial tendency of increased yield was observed. However, further research is needed to corroborate these trends in future productive stages, as this fruit crop is perennial. 

## Figures and Tables

**Table 1:** Fruit traits and yield of avocado grown using sustainable practices.

Practice	Fruit Weight (g)	Number of Fruits Per Plant	Yield (kg Plant^−1^)	Firmness ^z^ (N)	Soluble Solids (°Brix)
T1	245.35 ± 7.16 a	53.89 ± 7.18 a	13.21 ± 1.87 a	2.05 ± 0.05 b	9.49 ± 0.13 a
T2	249.21 ± 7.33 a	63.37 ± 5.24 a	15.91 ± 1.39 a	2.23 ± 0.05 a	9.51 ± 0.12 a
T3	241.08 ± 8.66 a	54.56 ± 3.80 a	13.08 ± 0.89 a	1.93 ± 0.04 b	9.41 ± 0.13 ab
T4	251.65 ± 7.71 a	50.10 ± 5.23 a	12.62 ± 1.45 a	1.98 ± 0.04 b	9.01 ± 0.11 b

T1 = fertigation, T2 = fertigation + microorganisms, T3 = microorganisms, T4 = control. ^z^ statistically significant differences found using Kruskal–Wallis test. Different letters indicate significant differences (*p* < 0.05) using ANOVA one-way analysis followed by Tukey’s test.

**Table 2:** Proximal analysis (DW) of avocado pulp grown using sustainable practices.

Practice	Dry Matter (%)	Moisture (%)	Ash (g 100 g^−1^)	Protein ^z^ (g 100 g^−1^)	Fiber (g 100 g^−1^)	Fat ^z^ (g 100 g^−1^)	Total Carbohydrates (g 100 g^−1^)
T1	32.24 ± 1.58 a	67.76 ± 1.58 a	10.11 ± 0.52 a	6.15 ± 0.20 ab	8.37 ± 0.23 a	70.71 ± 0.55 b	4.67 ± 0.37 a
T2	30.67 ± 1.47 a	69.33 ± 1.47 a	8.90 ± 0.39 a	6.49 ± 0.16 a	8.40 ± 0.58 a	72.66 ± 0.22 a	3.55 ± 0.70 a
T3	28.19 ± 0.66 a	71.81 ± 0.66 a	9.32 ± 0.77 a	6.00 ± 0.22 ab	7.88 ± 0.15 ab	70.98 ± 1.01 ab	5.82 ± 1.00 a
T4	29.31 ± 1.31 a	70.69 ± 1.31 a	10.44 ± 0.36 a	5.13 ± 0.33 b	7.24 ± 0.20 b	71.97 ± 0.37 ab	5.23 ± 0.46 a

T1 = fertigation, T2 = fertigation + microorganisms, T3 = microorganisms, T4 = control. ^z^ statistically significant differences found using Kruskal–Wallis test. Different letters indicate significant differences (*p* < 0.05) using ANOVA one-way analysis followed by Tukey’s test. DW = dry weight basis.

**Table 3:** Mineral content of avocado pulp (DW) grown using sustainable practices. * Values are expressed as mg 100 g pulp^−1^, ** values are expressed as g 100 g pulp^−1^.

Practice	Ca ^z^ *	P **	Mg *	K **	Na *	Cu ^z^ *	Fe ^z^ *	Mn ^z^ *	Zn ^z^ *
T1	54.67 ± 14.10 a	0.14 ± 0.01 a	96.90 ± 5.90 a	1.73 ± 0.07 a	8.70 ± 1.15 a	0.63 ± 0.04 a	1.53 ± 0.09 a	0.29 ± 0.03 a	2.43 ± 0.90 a
T2	36.03 ± 8.00 ab	0.13 ± 0.01 a	76.87 ± 5.67 b	1.51 ± 0.04 a	6.55 ± 1.11 a	0.72 ± 0.03 a	1.93 ± 0.57 a	0.25 ± 0.02 a	0.98 ± 0.16 b
T3	31.10 ± 3.08 ab	0.15 ± 0.01 a	72.58 ± 3.85 b	1.52 ± 0.12 a	9.25 ± 1.66 a	0.70 ± 0.03 a	2.15 ± 0.54 a	0.23 ± 0.02 a	1.95 ± 0.34 a
T4	26.35 ± 1.93 b	0.15 ± 0.01 a	68.40 ± 3.29 b	1.39 ± 0.11 a	6.22 ± 1.55 a	0.70 ± 0.03 a	3.70 ± 1.07 a	0.23 ± 0.02 a	1.52 ± 0.27 ab

T1 = fertigation, T2 = fertigation + microorganisms, T3 = microorganisms, T4 = control^. z^ statistically significant differences found using Kruskal–Wallis test. Different letters indicate significant differences (*p* < 0.05) using ANOVA one-way analysis followed by Tukey’s test. DW = dry weight basis.

**Table 4:** Antioxidant compounds and antioxidant capacity of avocado pulp (DW) grown using sustainable practices.

Practice	Polyphenols (mg GAE g^−1^)	Flavonoids (mg Catechin g^−1^)	Carotenoids (µg β Carotene g^−1^)	ABTS (µmol TE g^−1^)	FRAP (µmol TE g^−1^)
T1	8.74 ± 0.86 a	3.72 ± 0.13 a	52.60 ± 1.36 a	195.61 ± 17.21 a	131.04 ± 10.89 a
T2	10.25 ± 0.69 a	3.91 ± 0.05 a	49.89 ± 1.57 ab	209.37 ± 13.03 a	148.19 ± 11.00 a
T3	9.20 ± 0.52 a	3.54 ± 0.14 a	49.76 ± 2.09 ab	199.55 ± 6.23 a	135.28 ± 8.33 a
T4	9.27 ± 0.73 a	3.89 ± 0.15 a	45.03 ± 1.71 b	207.13 ± 8.56 a	142.02 ± 10.40 a

T1 = fertigation, T2 = fertigation + microorganisms, T3 = microorganisms, T4 = control. Different letters indicate significant differences (*p* < 0.05) using ANOVA one-way analysis followed by Tukey’s test. DW = dry weight basis.

**Table 5:** Fatty acid content of avocado pulp (FW) grown using sustainable practices. Results are expressed as percentages.

Practice	Palmitic	Palmitoleic	Stearic ^z^	Oleic	Linolelaidic	Linoleic	Linolenic
T1	19.83 ± 0.42 ab	9.11 ± 0.30 a	0.53 ± 0.01 a	50.20 ± 0.74 a	7.53 ± 0.49 a	11.97 ± 0.32 a	0.83 ± 0.03 a
T2	18.45 ± 0.43 b	8.63 ± 0.22 a	0.56 ± 0.05 ab	51.24 ± 0.46 a	7.72 ± 0.37 a	12.45 ± 0.27 a	0.95 ± 0.03 a
T3	20.04 ± 0.35 a	9.21 ± 0.21 a	0.50 ± 0.01 ab	50.47 ± 0.74 a	7.27 ± 0.44 a	11.83 ± 0.51 a	0.84 ± 0.04 a
T4	19.88 ± 0.55 ab	8.85 ± 0.37 a	0.49 ± 0.02 b	50.60 ± 1.17 a	7.14 ± 0.87 a	12.04 ± 0.47 a	0.87 ± 0.03 a

T1 = fertigation, T2 = fertigation + microorganisms, T3 = microorganisms, T4 = control. ^z^ statistically significant differences found using Kruskal–Wallis test. Different letters indicate significant differences (*p* < 0.05) using ANOVA one-way analysis followed by Tukey’s test. FW = fresh weight basis.

**Table 6:** Pearson correlation coefficients between the proximal variables and mineral elements.

	DM	Moisture	Ash	Fat	Protein	Fiber	TC	Ca	P	Mg	K	Na	Cu	Fe	Mn	Zn
**DM**	1.00	−0.99 **	0.03	0.13	0.17	0.37	−0.40	0.31	−0.40	0.19	0.11	−0.16	−0.22	0.20	−0.11	0.08
**Moisture**		1.00	−0.03	−0.13	−0.17	−0.37	0.40	−0.31	0.40	−0.19	−0.11	0.16	0.22	−0.20	0.11	−0.08
**Ash**			1.00	−0.36	−0.40	−0.16	−0.20	0.28	0.62 **	0.26	0.37	0.05	0.09	0.37	0.11	0.43
**Fat**				1.00	0.05	−0.15	−0.58 **	−0.32	−0.47	−0.37	−0.41	−0.58 **	0.10	−0.02	−0.19	−0.54 **
**Protein**					1.00	0.30	−0.31	−0.09	−0.22	0.17	0.04	0.06	0.07	−0.59 **	0.18	−0.07
**Fiber**						1.00	−0.38	0.02	−0.13	0.22	0.33	0.35	−0.09	−0.12	0.18	0.26
**TC**							1.00	0.10	0.11	−0.04	−0.10	0.28	−0.14	0.04	−0.08	0.05
**Ca**								1.00	0.09	0.65 **	0.25	0.28	−0.56 **	0.12	0.09	0.10
**P**									1.00	0.16	0.34	0.30	0.11	−0.01	0.25	0.48
**Mg**										1.00	0.74 **	0.26	−0.22	0.03	0.34	0.13
**K**											1.00	0.37	0.07	0.23	0.32	0.13
**Na**												1.00	−0.32	−0.09	0.13	0.13
**Cu**													1.00	0.06	0.35	−0.04
**Fe**														1.00	−0.05	0.08
**Mn**															1.00	0.24
**Zn**																1.00

DM = dry matter, TC = total carbohydrates. ** statistical differences at 1%.

**Table 7:** Pearson correlation coefficients between the antioxidant compounds and fatty acids.

	Polyphenol	FRAP	ABTS	Flavonoid	Carotenoid	Palmitic	Palmitoleic	Stearic	Oleic	Linolelaidic	Linoleic	Linolenic
**Polyphenol**	1.00	0.93 **	0.73 **	0.63 **	−0.03	−0.44	−0.04	−0.18	−0.06	0.50 **	0.36	0.43
**FRAP**		1.00	0.62 **	0.62 **	−0.09	−0.33	0.16	−0.16	−0.18	0.48	0.34	0.38
**ABTS**			1.00	0.41	0.01	−0.26	−0.13	−0.13	0.01	0.17	0.26	0.37
**Flavonoid**				1.00	−0.11	−0.51 **	−0.22	−0.19	−0.04	0.54 **	0.50 **	0.60 **
**Carotenoid**					1.00	0.11	−0.02	0.15	−0.25	0.14	0.27	−0.04
**Palmitic**						1.00	0.58 **	0.06	−0.55 **	−0.59 **	−0.14	−0.59 **
**Palmitoleic**							1.00	0.13	−0.73 **	0.06	0.02	−0.30
**Stearic**								1.00	0.04	−0.13	−0.21	−0.15
**Oleic**									1.00	−0.28	−0.62 **	−0.14
**Linolelaidic**										1.00	0.59 **	0.58 **
**Linoleic**											1.00	0.79 **
**Linolenic**												1.00

** statistical differences at 1%.

## Data Availability

The data are contained within the article.
